# Nebivolol Protects the Thoracic and Abdominal Aorta and Their Perivascular Adipose Tissue From the Differential Detrimental Effects of Obesity

**DOI:** 10.1111/fcp.70096

**Published:** 2026-06-15

**Authors:** Thales M. H. Dourado, Gustavo F. Pimenta, Leonardo P. Silva, Victor O. Assis, Alessandra O. Silva, Carlos R. Tirapelli

**Affiliations:** ^1^ Programa de Pós‐Graduação em Farmacologia, Faculdade de Medicina de Ribeirão Preto Universidade de São Paulo (USP) Ribeirão Preto São Paulo Brazil; ^2^ Laboratório de Farmacologia Cardiovascular, Departamento de Ciências BioMoleculares, Faculdade de Ciências Farmacêuticas de Ribeirão Preto USP Ribeirão Preto São Paulo Brazil

**Keywords:** abdominal, aorta, nebivolol, obesity, PVAT, thoracic

## Abstract

The effects of obesity on the vasculature and perivascular adipose tissue (PVAT) function vary according to the vascular territory. Nebivolol is a third‐generation β‐blocker that exerts vasculoprotective effects in obesity. The thoracic and abdominal aorta show different responses under physiological conditions, and their PVATs have a distinctive composition and secretory profile. We hypothesized that obesity would affect thoracic and abdominal aortic responsiveness, as well as their PVAT function, differently and that nebivolol would restore obesity‐induced vascular and PVAT changes. To test this hypothesis, male Wistar Hannover rats were fed a hypercaloric diet for 14 weeks. Nebivolol (10 mg/kg/day) was administered via gavage during the last 4 weeks. Obesity increased superoxide (O_2_•^−^) generation within the thoracic PVAT, but this response did not result in a loss of its anticontractile effect. Vascular hypercontractility was detected in thoracic aortas, which was mediated by the overproduction of NADPH oxidase‐derived O_2_•^−^ and reduced nitric oxide (NO) bioavailability. Conversely, obesity induced a pro‐contractile phenotype in the PVAT of the abdominal aorta. This response occurred in an endothelium‐dependent manner, was ameliorated by tiron, and was accompanied by increased O_2_•^−^ levels. In all cases, nebivolol reversed the effects of obesity. In summary, obesity differentially affects the thoracic and abdominal aorta and their respective PVATs. Nebivolol exerts vasculoprotective effects through antioxidant mechanisms, leading to a reversal of obesity‐induced vascular hypercontractility in both aortic segments. The beneficial effects of nebivolol described here could help mitigate cardiovascular complications associated with obesity. Obesity induces PVAT dysfunction. Extending this knowledge, we demonstrated that obesity differentially affects the thoracic and abdominal aorta along with their associated PVATs. This widespread impairment of PVAT function can drive various adverse vascular consequences, including arterial hypertension and aneurysm formation. Critically, peri‐aortic PVAT is of particular clinical relevance due to its association with metabolic risk factors and vascular calcification. Nebivolol exerts vasculoprotective effects via antioxidant mechanisms within PVAT, ultimately reversing obesity‐induced vascular hypercontractility in both aortic segments. Thus, investigating pharmacological agents that restore PVAT function, as demonstrated here with nebivolol, represents a promising therapeutic strategy to mitigate obesity‐related cardiovascular complications.

## 1 Introduction

The detrimental effects of obesity on the cardiovascular system are associated with cardiometabolic changes, including insulin resistance and alterations in the lipid profile such as elevated plasma concentrations of triglycerides and low‐density lipoproteins (LDL) [1, 2]. Obesity is also linked to arterial hypertension. This condition is often managed with antihypertensive drugs that can counteract the insulin resistance induced by obesity [3]. Third‐generation β‐blockers exert beneficial metabolic and endocrine effects, making them suitable for obese patients with arterial hypertension [2]. Nebivolol is a third‐generation β‐blocker that lowers insulin resistance and reduces circulating concentrations of cholesterol, LDL, and triglycerides [2, 4]. In addition, the drug provides vascular and cardiac protection, improving the prognosis of cardiovascular diseases [5]. The antihypertensive effect of nebivolol occurs primarily through selective blockade of β_1_‐adrenoreceptors. However, it also displays additional actions, including antioxidant and anti‐inflammatory effects, as well as activation of β_3_‐adrenoreceptors [6].

Obesity promotes pro‐oxidative and pro‐inflammatory responses in the vasculature that result in endothelial dysfunction and vascular hypercontractility ([7]; Muñoz et al. 2013). Nebivolol exerts vasculoprotective effects in obesity that occur mainly through antioxidant and anti‐inflammatory actions. The antioxidant effects of nebivolol include the reduction of NADPH oxidase activity and superoxide (O_2_•^−^) generation [8, 9], while its anti‐inflammatory actions in the vasculature occur through reduction of the expression of adhesion molecules (e.g., VCAM‐1 and ICAM‐1) and pro‐inflammatory cytokines (e.g., TNF‐α and IL‐1β), and the mitigation of macrophage infiltration [9, 10, 11].

The harmful effects of obesity are also described in the perivascular adipose tissue (PVAT). Physiologically, PVAT regulates vascular tone by secreting a variety of relaxing molecules [e.g., nitric oxide (NO), adiponectin, and prostaglandin] that exert anticontractile effects [12]. Obesity disrupts the anticontractile action of PVAT through multiple mechanisms, such as the overproduction of reactive oxygen species (ROS) by NADPH oxidase, reduction of NO bioavailability, and decrease in antioxidant capacity [13, 14, 15]. As a result of these changes, PVAT becomes dysfunctional and starts contributing to vascular and endothelial dysfunction.

The cellular composition of PVAT varies according to vascular territory. For example, the PVAT surrounding the abdominal aorta [(a)PVAT] possesses a predominant number of unilocular white‐like adipocytes and a small proportion of brown‐like adipocytes, while PVAT from the thoracic aorta [(t)PVAT] is mostly composed of brown‐like adipocytes [16]. Differences in composition are directly related to changes in the secretory profile and anticontractile action of PVAT [12, 17]. In this sense, it has been shown that (a)PVAT does not exert anticontractile effects under physiological conditions, whereas (t)PVAT exerts an anticontractile effect that is mainly mediated by NO [17, 18].

The effects of obesity on the vascular system are vessel‐specific. Similarly, obesity differentially alters PVAT function, a response attributable to PVAT heterogeneity [19]. We hypothesized that (1) obesity would differentially affect thoracic and abdominal aorta responsiveness as well as the function of their associated PVAT, and (2) nebivolol would be able to restore the vascular and PVAT changes promoted by obesity. The thoracic and abdominal aorta were chosen due to their different physiological responses and the heterogeneity in their PVAT composition [12, 17]. Here, we aimed to evaluate whether nebivolol would restore the effects of obesity in different vascular territories and to investigate the possible mechanisms underlying this response.

## 2 Material and Methods

### 2.1 Animal Housing and Treatment

The vasculoprotective effect of nebivolol on obesity‐induced vascular changes was studied in male Wistar Hannover rats. Initially weighing 230–260 g (50–60 days old), the rats (*n* = 120) were fed a highly palatable hypercaloric diet for 14 weeks, with the following composition by weight: ground standard chow (33%), Nestlé condensed milk (3%), sucrose (7%), and water (27%). The obese group received free access to this diet along with filtered water containing sucrose (100 mg/mL), whereas the control group received standard chow and filtered water without sucrose. From weeks 10 to 14, the nebivolol group (fed the standard chow) and a separate obese‐nebivolol group (fed the hypercaloric diet) received nebivolol (10 mg/kg/day) via gavage, solubilized in saline containing 0.25% carboxymethyl cellulose. To control for the administration procedure, rats in the control and obese groups received the vehicle (0.25% carboxymethyl cellulose, 10 mL/kg/day) by gavage during the same period.

The composition and duration of the hypercaloric diet exposure were chosen based on previous findings demonstrating cardiometabolic and vascular alterations in male rats [20, 21, 22, 23]. The diet used in the present study leads to an increase in blood pressure, insulin resistance, alterations in the lipid profile (e.g., reduced HDL levels and elevated plasma triglycerides, total cholesterol, and LDL levels), and changes in anthropometric parameters [8]. The dose and administration regimen of nebivolol were selected based on prior studies demonstrating its vasculoprotective effects [8, 24]. Treatment with nebivolol began at week 10, as metabolic and vascular changes were observed in rats on the hypercaloric diet by this time [13].

Following the 14‐week experimental period, the rats were deeply anesthetized with an intraperitoneal injection of urethane (1.25 g/kg; Cat. No. U2500, Sigma‐Aldrich, St. Louis, MO, USA). While under deep anesthesia, the animals were euthanized by exsanguination and diaphragm rupture. Death was confirmed by the absence of respiration, loss of reflexes, and the presence of fixed, dilated pupils. The thoracic and abdominal aortas, together with their associated PVAT, were subsequently dissected for functional and biochemical analyses. All tissues were collected, snap‐frozen in liquid nitrogen, and stored at −80°C until use.

### 2.2 Vascular Reactivity Assays

Functional assays were conducted using thoracic and abdominal aortic rings, which were prepared either with or without PVAT and with either an intact or mechanically denuded endothelium. The thoracic and abdominal aortas were isolated and cut into 5‐mm‐long rings. These rings were then mounted in 5‐mL organ baths filled with Krebs solution (37°C; composition in mmol/L: NaCl 118.0, KCl 4.7, KH_2_PO_4_ 1.2, MgSO_4_ 1.2, NaHCO_3_ 15.0, Glucose 5.5, and CaCl_2_ 2.5), continuously gassed with carbogen (95% O_2_, 5% CO_2_). The rings were maintained at a basal tension of 15 mN for a 60‐min equilibration period. Following equilibration, the rings were stimulated twice with phenylephrine (0.1 μmol/L) to assess maximum contractility, then washed and allowed to return to baseline tension. After a further 30 min, cumulative concentration‐response curves to phenylephrine (0.0001 to 10 μmol/L; Sigma‐Aldrich, Cat. No. 61‐76‐7) were obtained. In experiments assessing the role of the endothelium, it was removed by gently rolling the luminal surface of the ring over a wire. Successful denudation was confirmed by the absence of a relaxation response to acetylcholine (0.1 μmol/L) in rings pre‐contracted with phenylephrine. For all concentration‐response curves, the maximum effect (E_max_), the negative logarithm of the half‐maximal effective concentration (pD_2_) were calculated by nonlinear regression analysis using GraphPad Prism 8.02 (GraphPad Software Inc., San Diego, CA, USA), which was also used to calculate the area under the curve (AUC).

To assess the contribution of ROS to obesity‐induced vascular dysfunction, thoracic aortas (with or without PVAT) were incubated for 30 min with the O_2_•^−^ scavenger tiron (1 mmol/L; Sigma‐Aldrich, Cat. No. 270573‐71‐2). The effect of obesity on NO bioavailability was investigated in endothelium‐intact aortas (with or without PVAT) following a 30 min incubation with the nonselective NOS inhibitor L‐NAME (100 μmol/L; Sigma‐Aldrich, Cat. No. 51298–62‐5). Both tiron and L‐NAME were dissolved in distilled water; concentrations were chosen based on previous studies [25].

### 2.3 Determination of Oxidative Parameters

The lucigenin‐derived chemiluminescence assay was used to determine the generation of O_2_•^−^ by NADPH oxidase as described by Yogi et al. [26]. Briefly, the aorta or PVAT was homogenized in phosphate‐buffered saline (PBS; pH 7.4). The homogenate (50 μL) was transferred to a white microplate containing 5 μmol/L lucigenin (Bis‐N‐methylacridinium nitrate; Cat. No. 2315–97‐1, Sigma‐Aldrich). The microplate was placed in a luminometer (Orion II luminometer, Berthold Detection Systems, Germany) for a basal reading. Then, 0.1 mmol/L NADPH (Cat. No. 2646‐71‐1, Sigma‐Aldrich), the substrate for NADPH oxidase, was added to the samples, and the microplate was placed in the luminometer for a second reading. The luminescence obtained in the second reading was subtracted from the basal values measured in the absence of NADPH. The results are expressed as relative light units (RLU)/mg of protein.

To determine the total SOD activity, the aorta or PVAT was homogenized in PBS (pH 7.4) using a glass homogenizer. The samples were centrifuged (10 000 × g, 10 min, 4°C), and 20 μL of the supernatant was transferred to a 96‐well microplate. SOD activity was measured at 450 nm according to the instructions of a commercially available kit (Cat. No. 19160, Sigma‐Aldrich). The results are expressed as percentage (%) inhibition rate/mg of protein.

The concentration of malondialdehyde (MDA) in the aorta and PVAT was determined colorimetrically as previously described by Dourado et al. [27]. Briefly, tissues were homogenized in Tris–HCl buffer (pH 7.4) and centrifuged (1600 × g, 10 min, 4°C). The supernatants were incubated at 95°C for 60 min with a solution containing Milli‐Q water, 8.1% sodium dodecyl sulfate (SDS), acetic acid (pH 3.5), and 0.6% thiobarbituric acid. After incubation, the samples were centrifuged again (1600 × g, 10 min, 4°C), and the absorbance of the supernatants was measured at 532 nm to determine the MDA concentration. The results are expressed as mmol/mg of protein.

### 2.4 Determination of Nitrite Concentration

To determine nitrite concentration, the aorta or PVAT was homogenized in PBS (pH 7.4) and centrifuged (10 000 × g, 20 min, 4°C). A 50 μL aliquot of the supernatant was incubated with Griess reagent (5% phosphoric acid, 0.2% n‐(1‐naphthyl)ethylenediamine dihydrochloride, and 2% sulfanilamide) for 10 min at room temperature. Absorbance was measured at 540 nm, and nitrite concentration was calculated using a nitrite standard curve (0–200 μmol/L) according to Dourado et al. [25]. Results are expressed as μmol/mg of protein.

### 2.5 Statistical Analysis

Results are presented as means ± standard error of the mean (S.E.M.). A two‐way ANOVA followed by Tukey's post hoc test was used to detect possible diferences among groups. A significance level of *p* < 0.05 was set. The software GraphPad Prism version 8.02 (San Diego, CA, USA) was used to conduct the statistical analyses. Sample size was determined using the software G*Power (University of Kent, Canterbury, England).

## 3 Results

### 3.1 Effects of Obesity and Nebivolol on Thoracic Aorta and PVAT

**FIGURE 1:**
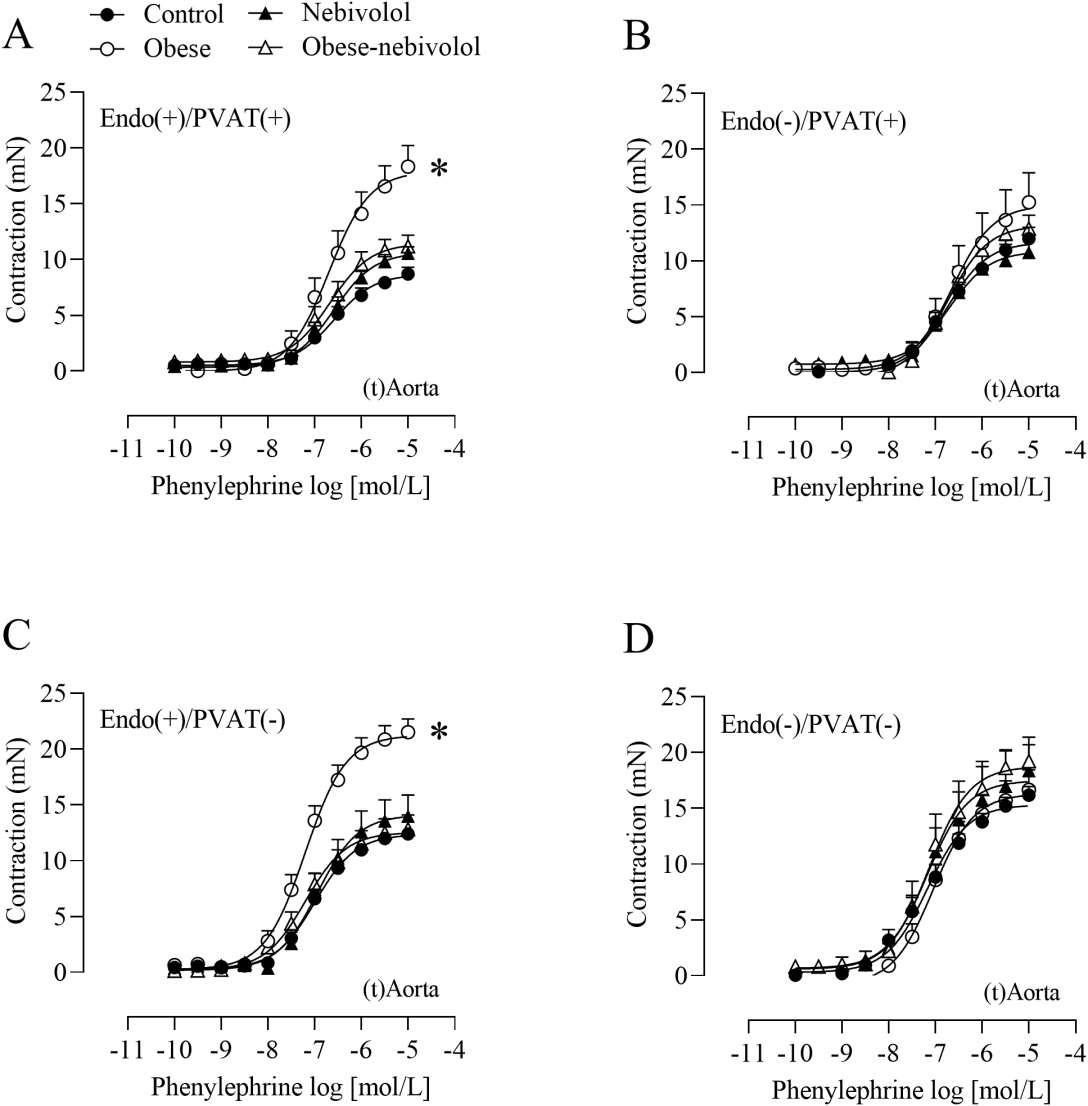
Nebivolol reverses the vascular hypercontractility induced by obesity in the thoracic aorta. Concentration‐response curves to phenylephrine were determined in the thoracic aorta [(t)Aorta] with intact [Endo(+)] or denuded endothelium [Endo(−)] in the presence [PVAT(+)] or absence of PVAT [PVAT(−)] (A–D). Values are given as mean ± SEM of *n* = 5–9 rats. *Compared to the control, nebivolol and obese‐nebivolol (*p* < 0.05, two‐way ANOVA followed by Tukey's post hoc test). The asterisk (*) denotes significant differences in either Emax or pD_2_ values (provided in Table 1).

In thoracic aortas from control rats, PVAT attenuated the contractile response induced by phenylephrine in both endothelium‐intact and endothelium‐denuded preparations (Table 1). Obesity increased the contractile response to phenylephrine in endothelium‐intact, but not endothelium‐denuded aortic rings with PVAT. Treatment with nebivolol fully reversed obesity‐induced vascular hypercontractility (Figure 1A,B; Tables 1 and 3). Similar results were found in arteries without PVAT, where nebivolol reversed the obesity‐induced increase in phenylephrine‐induced contraction of endothelium‐intact, but not endothelium‐denuded arteries (Figure 1C,D; Tables 1 and 3).

**TABLE 1:** Effect of nebivolol on the E_max_ and pD_2_ values for phenylephrine in the thoracic or abdominal aorta of obese rats.

	Control		Obese	
Groups	Thoracic aorta			
	E_max_ (mN)	pD_2_	E_max_ (mN)	pD_2_
Control E(+) /PVAT(+)	8.7 ± 0.6 (9)	6.6 ± 0.1	18.3 ± 1.9 (8)^a^	6.7 ± 0.1
Control E(+) /PVAT(−)	12.4 ± 0.5 (9)	7.0 ± 0.1	21.5 ± 1.1 (9)^a^	7.2 ± 0.1
Control E(−) /PVAT(+)	11.9 ± 1.0 (9)	6.7 ± 0.1	15.2 ± 2.6 (8)	6.6 ± 0.2
Control E(−) /PVAT(−)	16.1 ± 0.7 (9)	7.1 ± 0.1	16.6 ± 1.7 (9)	7.0 ± 0.1
Nebivolol E(+) /PVAT(+)	10.5 ± 0.6 (9)	6.7 ± 0.1	11.2 ± 1.0 (7)	6.7 ± 0.1
Nebivolol E(+) /PVAT(−)	13.8 ± 1.2 (6)	7.2 ± 0.1	14.0 ± 1.9 (6)	6.9 ± 0.1
Nebivolol E(−) /PVAT(+)	12.8 ± 1.2 (6)	6.7 ± 0.1	10.7 ± 1.5 (6)	6.7 ± 0.1
Nebivolol E(−) /PVAT(−)	19.2 ± 1.5 (9)	7.1 ± 0.1	18.4 ± 3.0 (6)	7.2 ± 0.2

	Abdominal aorta			
	E_max_ (mN)	pD_2_	E_max_ (mN)	pD_2_
Control E(+) /PVAT(+)	10.8 ± 1.3 (9)	6.7 ± 0.2	19.9 ± 3.6 (6)^a^	6.4 ± 0.2
Control E(+) /PVAT(−)	11.4 ± 1.1 (9)	6.8 ± 0.1	12.3 ± 1.5 (7)	6.7 ± 0.1
Control E(−) /PVAT(+)	12.9 ± 1.2 (7)	6.8 ± 0.1	13.4 ± 1.2 (9)	6.5 ± 0.1
Control E(−) /PVAT(−)	15.6 ± 2.5 (6)	6.8 ± 0.1	15.4 ± 2.8 (9)	6.7 ± 0.1
Nebivolol E(+) /PVAT(+)	11.5 ± 1.5 (7)	6.7 ± 0.1	10.0 ± 1.2 (6)	6.7 ± 0.1
Nebivolol E(+) /PVAT(−)	14.1 ± 1.5 (9)	7.0 ± 0.1	11.6 ± 1.2 (8)	6.8 ± 0.1
Nebivolol E(−) /PVAT(+)	10.8 ± 1.0 (9)	6.8 ± 0.1	10.6 ± 1.4 (7)	6.6 ± 0.1
Nebivolol E(−) /PVAT(−)	12.5 ± 0.6 (9)	7.0 ± 0.1	14.1 ± 2.1 (7)	6.8 ± 0.1

*Note:* The number (n) of animals used in each set of experiments is indicated after each set of measurements. Values are given as means ± SEM.

^a^ Compared to the respective control, nebivolol and obese‐nebivolol group.

^b^ Compared to the obese group (*p* < 0.05; two‐way ANOVA followed by Tukey's post hoc test).

**FIGURE 2:**
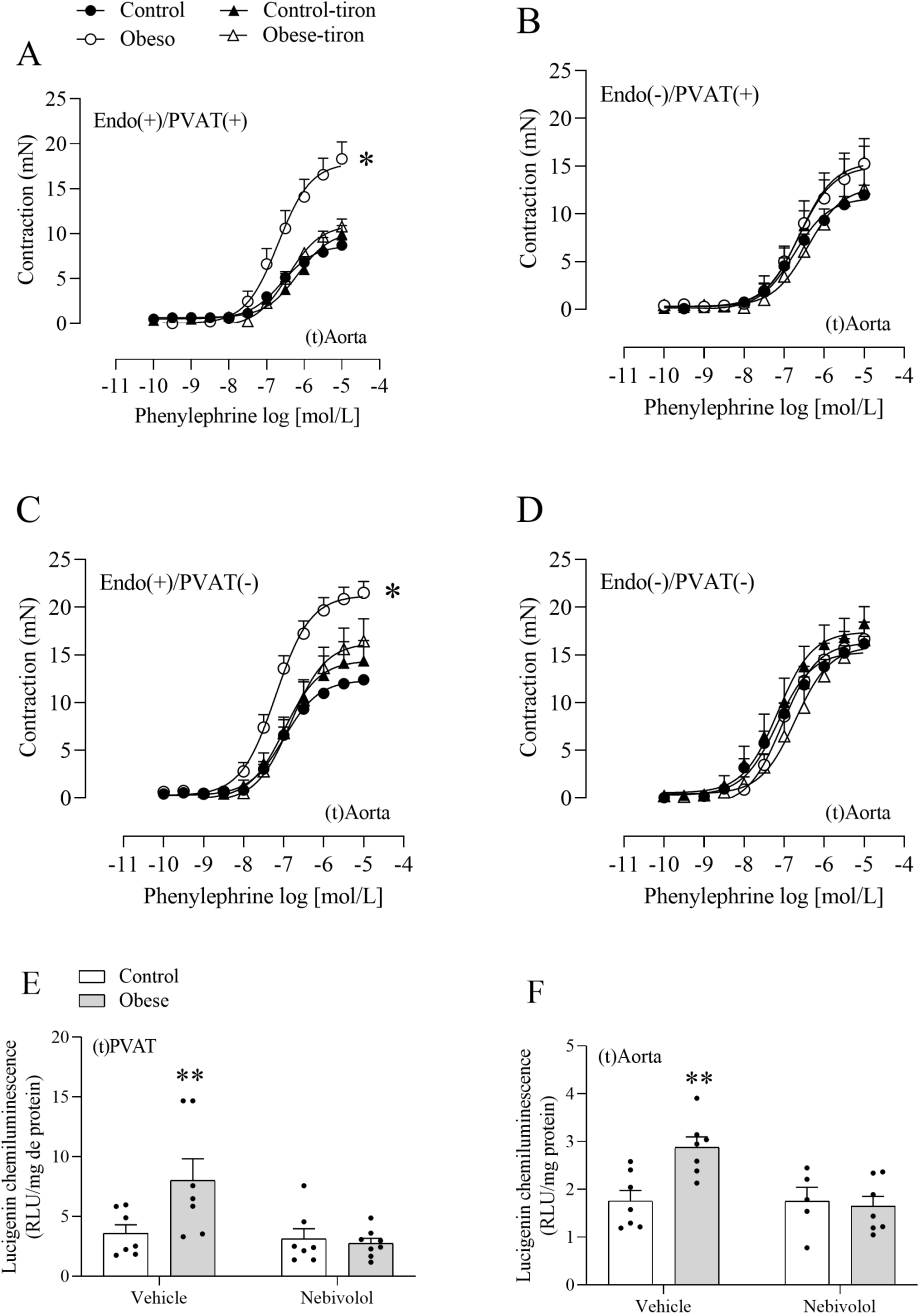
Tiron reverses obesity‐induced vascular hypercontractility in the thoracic aorta. Concentration‐response curves to phenylephrine were obtained in the thoracic aorta [(t)Aorta] with intact [Endo(+)] or denuded endothelium [Endo(−)] in the presence [PVAT(+)] or absence of PVAT [PVAT(−)] after incubation (30 min) with tiron (1 mmol/L) (A–D). NADPH oxidase‐derived O_2_•^−^ was measured in the (t)Aorta and its PVAT [(t)PVAT] using the lucigenin chemiluminescence assay (E, F). Values are given as means ± SEM of *n* = 5–9 rats. *Compared to the control, control‐tiron, and obese‐tiron groups;**Compared to the control, nebivolol and obese‐nebivolol groups (*p* < 0.05; two‐way ANOVA followed by Tukey's post hoc test). The symbols * and ** denote differences in the E_max_ or pD_2_ values (provided in Table 2).

Given that oxidative stress is a central mechanism whereby obesity alters the vascular function, we assessed its contribution to the pro‐contractile effect of obesity. The O_2_•^−^ scavenger tiron reversed obesity‐induced hypercontractility to phenylephrine in endothelium‐intact aortas with PVAT, while it did not alter the contraction to phenylephrine in arteries without endothelium (Figure 2A,B; Tables 2 and 4). Similarly, tiron mitigated the hypercontractility induced by obesity in arteries with intact endothelium in the absence of PVAT (Figure 2C; Tables 2 and 4). In endothelium‐denuded arteries without PVAT, no changes in phenylephrine‐induced contraction were observed after incubation with tiron (Figure 2D; Tables 2 and 4). Both the thoracic aorta and PVAT from obese rats exhibited increased NADPH oxidase‐derived O_2_•^−^ production, which was reversed by nebivolol (Figure 2E,F).

**TABLE 2:** Effects of tiron or L‐NAME on the E_max_ and pD_2_ for phenylephrine in the thoracic or abdominal aorta of obese rats.

	Control		Obese	
Groups	Thoracic aorta			
	E_max_ (mN)	pD_2_	E_max_ (mN)	pD_2_
Control E(+) /PVAT(+)	8.7 ± 0.6 (9)	6.6 ± 0.1	18.3 ± 1.9 (8)^a^	6.7 ± 0.1
Control E(+) /PVAT(−)	12.4 ± 0.5 (9)	7.0 ± 0.1	21.5 ± 1.1 (9)^a^	7.2 ± 0.1
Control E(−) /PVAT(+)	11.9 ± 1.0 (9)	6.7 ± 0.1	15.2 ± 2.6 (8)	6.6 ± 0.2
Control E(−) /PVAT(−)	16.1 ± 0.7 (9)	7.1 ± 0.1	16.6 ± 1.7 (9)	7.0 ± 0.1
Tiron E(+) /PVAT(+)	9.9 ± 0.9 (9)	6.1 ± 0.1	10.7 ± 0.8 (7)^b^	6.4 ± 0.1
Tiron E(+) /PVAT(−)	14.3 ± 2.1 (8)	7.0 ± 0.1	16.2 ± 0.9 (6)^b^	6.8 ± 0.1
Tiron E(−) /PVAT(+)	15.5 ± 1.5 (9)	6.6 ± 0.1	12.6 ± 2.3 (6)	6.4 ± 0.1
Tiron E(−) /PVAT(−)	18.3 ± 1.7 (8)	7.1 ± 0.1	16.5 ± 1.9 (9)	6.7 ± 0.1
L‐NAME E(+) /PVAT(+)	14.6 ± 0.7 (5)^a^	7.1 ± 0.1	15.4 ± 0.5 (6)	6.9 ± 0.1
L‐NAME E(+) /PVAT(−)	17.2 ± 0.5 (7)^a^	7.3 ± 0.1	18.5 ± 0.9 (5)	6.9 ± 0.1

	Abdominal aorta			
	E_max_ (mN)	pD_2_	E_max_ (mN)	pD_2_
Control E(+) /PVAT(+)	10.8 ± 1.3 (9)	6.7 ± 0.2	19.9 ± 3.6 (6)^a^	6.4 ± 0.2
Control E(+) /PVAT(−)	11.4 ± 1.1 (9)	6.8 ± 0.1	12.3 ± 1.5 (7)	6.7 ± 0.1
Control E(−) /PVAT(+)	12.9 ± 1.2 (7)	6.8 ± 0.1	13.4 ± 1.2 (9)	6.5 ± 0.1
Control E(−) /PVAT(−)	15.6 ± 2.5 (6)	6.8 ± 0.1	15.4 ± 2.8 (9)	6.7 ± 0.1
Tiron E(+) /PVAT(+)	10.5 ± 1.3 (8)	6.7 ± 0.2	12.0 ± 1.1 (6)^b^	6.2 ± 0.1
Tiron E(+) /PVAT(−)	13.2 ± 1.5 (7)	6.8 ± 0.1	12.6 ± 1.1 (6)	6.6 ± 0.1
Tiron E(−) /PVAT(+)	11.2 ± 1.6 (6)	6.8 ± 0.1	12.7 ± 1.4 (7)	6.8 ± 0.1
Tiron E(−) /PVAT(−)	14.2 ± 2.4 (7)	6.9 ± 0.1	11.7 ± 1.0 (6)	6.8 ± 0.1

*Note:* The number (n) of animals used in each set of experiments is indicated after each set of measurements. Values are given as means ± SEM.

^a^ Compared to the control group.

^b^ Compared to the obese group in the absence of the inhibitor (*p* < 0.05; two‐way ANOVA followed by Tukey's post hoc test).

**FIGURE 3:**
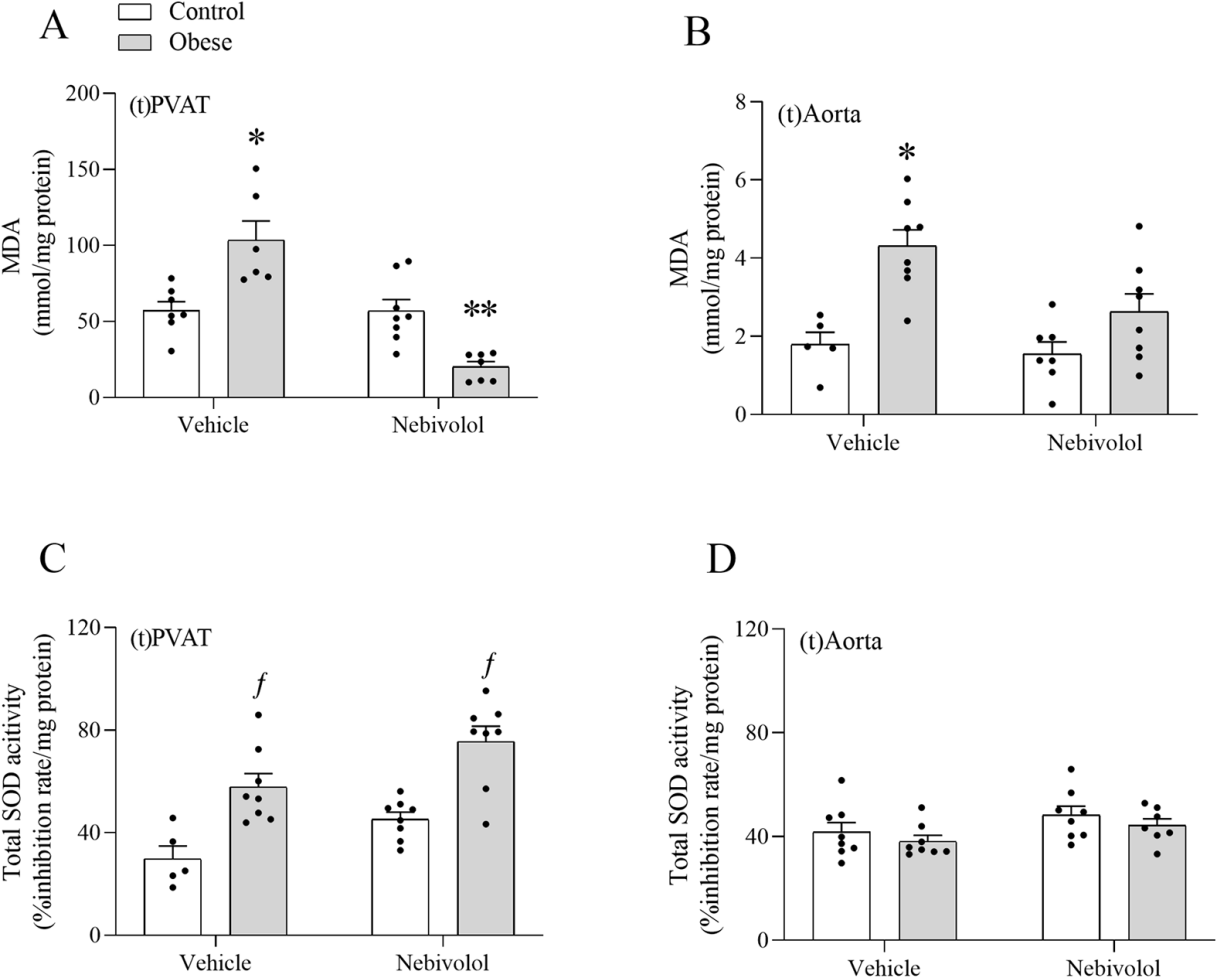
Effects of obesity on total SOD activity and lipoperoxidation in the thoracic aorta and thoracic PVAT. The concentration of MDA (A, B) and the activity of SOD (C, D) were determined colorimetrically. Values are given as means ± SEM of *n* = 5–8 rats. *Compared to the control, nebivolol, and obese‐nebivolol groups;**Compared to the control, obese and nebivolol groups;ƒCompared to the control and nebivolol groups (*p* < 0.05; two‐way ANOVA followed by Tukey's post hoc test).

Obesity increased the concentration of MDA in the thoracic aorta and PVAT, and nebivolol reversed this response (Figure 3A,B). Increased SOD activity was observed in the PVAT, but not in the aorta from obese rats. However, treatment with nebivolol failed to reverse this response (Figure 3C,D).

**FIGURE 4:**
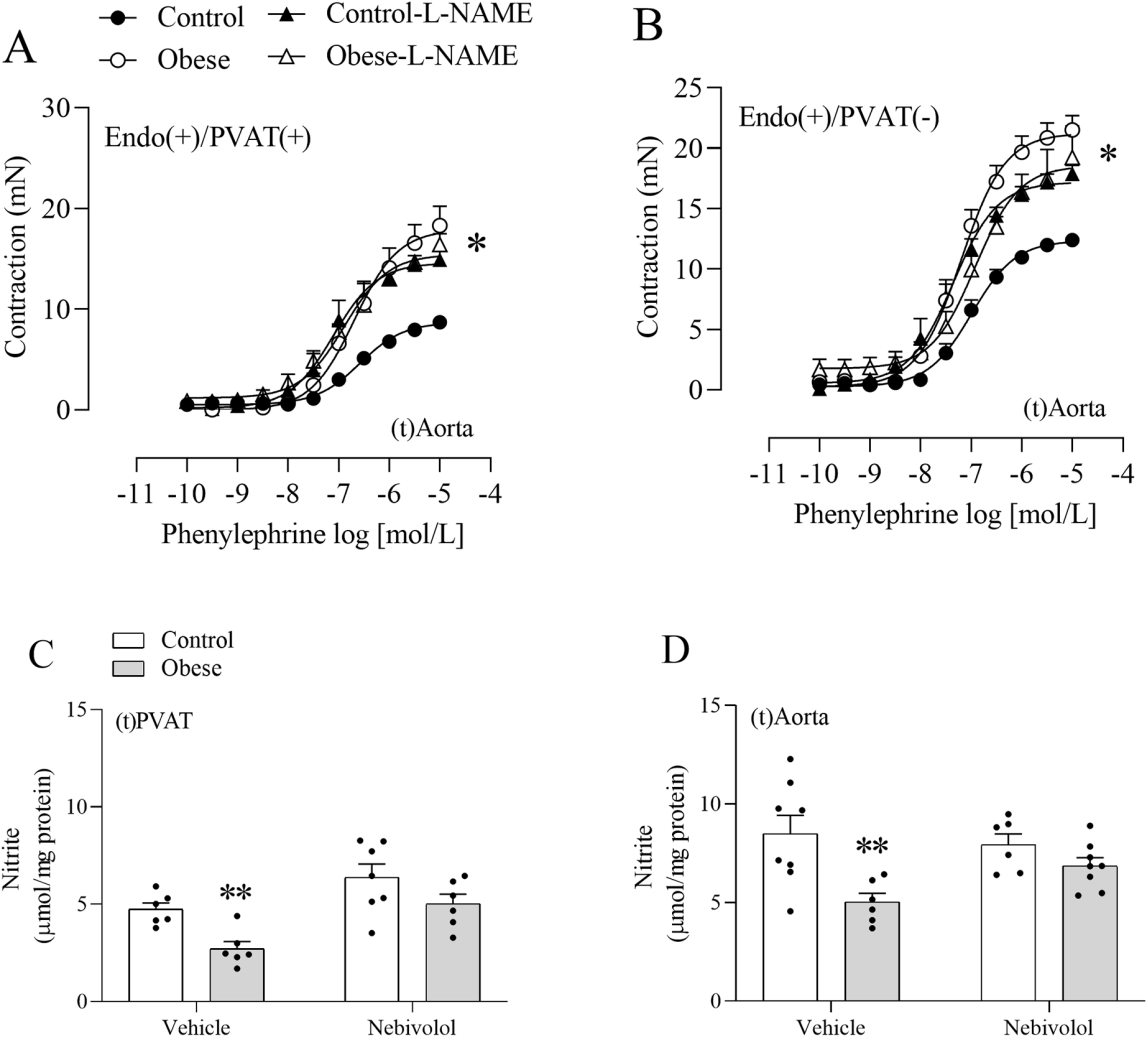
Effects of L‐NAME on obesity‐induced vascular hypercontractility in the thoracic aorta. Concentration‐response curves to phenylephrine were obtained in the thoracic aorta [(t)Aorta] with intact endothelium [Endo(+)] in the presence [PVAT(+)] or absence of PVAT [PVAT(−)] following a 30‐min incubation with L‐NAME (100 μmol/L) (A, B). The concentration of nitrite in the (t)Aorta or thoracic PVAT [(t)PVAT] was determined colorimetrically (C, D). Values are given as means ± SEM of *n* = 5–9 rats. *Compared to the control group;**Compared to the control, nebivolol and obese‐nebivolol groups (*p* < 0.05; two‐way ANOVA followed by Tukey's post hoc test). The asterisk (*) denotes significant differences in either E_max_ or pD_2_ values (provided in Table 2).

The interaction between O_2_•^−^ and NO may reduce NO bioavailability. Given that NO is an important vasodilator in the aorta, we evaluated whether obesity alters NO levels. L‐NAME, a nonselective NOS inhibitor, increased phenylephrine‐induced contraction in endothelium‐intact aortas with PVAT from control rats. This response, however, was not observed in aortas from obese rats (Figure 4A; Tables 2 and 4). Similar findings were obtained in endothelium‐intact aortas without PVAT, where L‐NAME failed to enhance contraction in rings from obese rats (Figure 4B; Tables 2 and 4). Nitrite concentrations were reduced in the thoracic aorta and PVAT of obese rats, an effect that was reversed by nebivolol (Figure 4C,D).

### 3.2 Effects of Obesity and Nebivolol on Abdominal Aorta and PVAT

**FIGURE 5:**
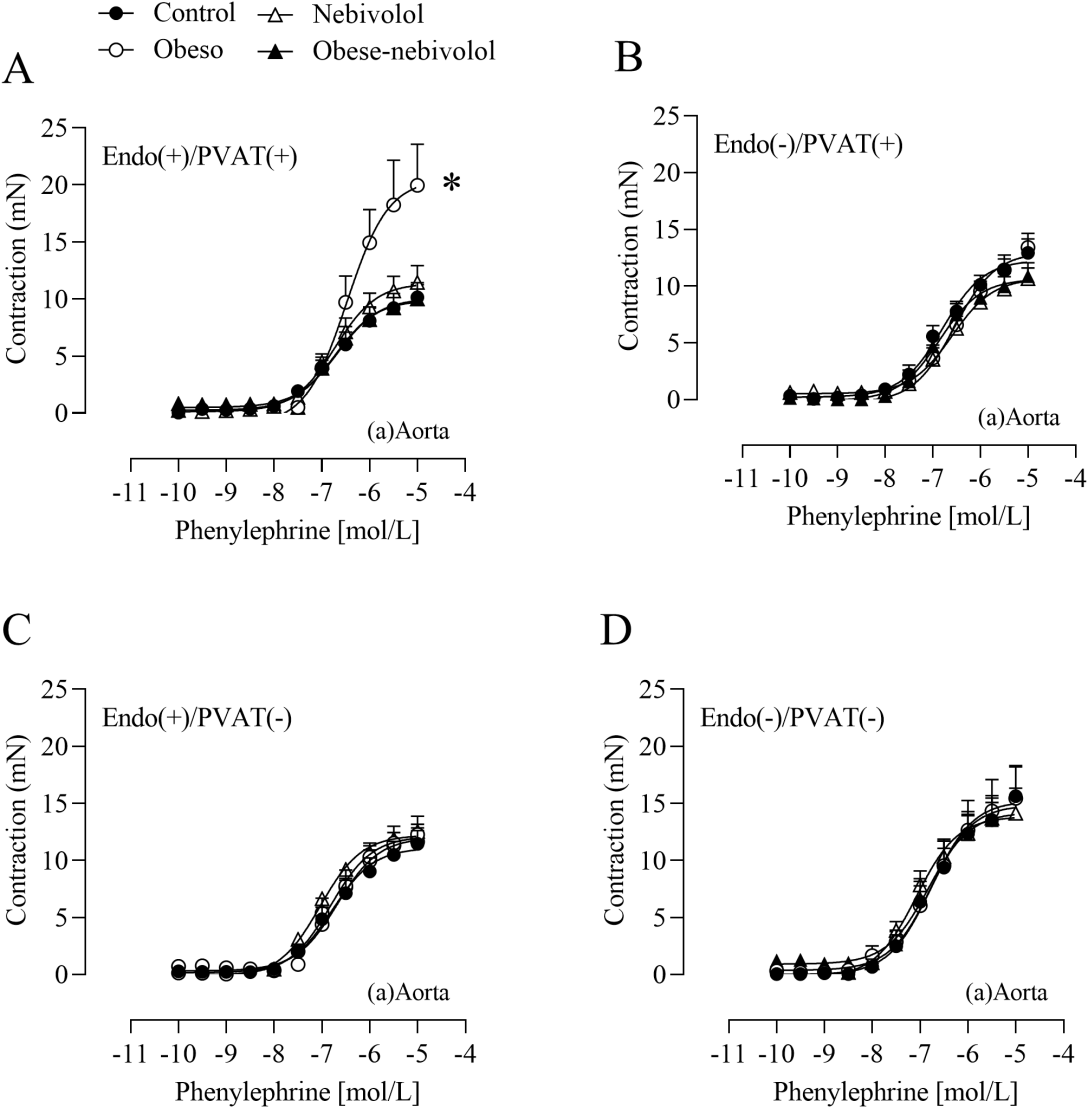
Nebivolol reverses obesity‐induced PVAT dysfunction in the abdominal aorta. Concentration‐response curves to phenylephrine were determined in the abdominal aorta [(a) Aorta] with intact [Endo(+)] or denuded endothelium [Endo(−)] in the presence [PVAT(+)] or absence of PVAT [PVAT(−)] (A–D). Values are given as mean ± SEM of *n* = 6–9 rats. *Compared to the control, nebivolol and obese‐nebivolol (*p* < 0.05, two‐way ANOVA followed by Tukey's post hoc test). The asterisk (*) denotes significant differences in either E_max_ or pD_2_ values (provided in Table 1).

Obesity increased phenylephrine‐induced contraction in endothelium‐intact abdominal aortas with PVAT, but not in those that were endothelium‐denuded. This hypercontractility to phenylephrine was reversed by nebivolol (Figure 5A,B; Tables 1 and 3). Conversely, obesity did not alter the contractile response to phenylephrine in aortas without PVAT, regardless of whether the endothelium was intact or denuded (Figure 5C,D; Tables 1 and 3).

**TABLE 3:** Area under the curve (AUC) of the concentration response curves for phenylephrine in the thoracic or abdominal aorta of control and obese rats.

Thoracic aorta		
Groups	Control	Obese
Control E(+) /PVAT(+)	15.5 ± 1.6 (9)	30.2 ± 4.2 (8)^a^
Control E(+) /PVAT(−)	25.3 ± 2.0 (9)	47.2 ± 3.4 (9)^a^
Control E(−) /PVAT(+)	20.4 ± 2.1 (9)	25.3 ± 5.2 (8)
Control E(−) /PVAT(−)	33.9 ± 3.0 (9)	32.8 ± 3.8 (9)
Nebivolol E(+) /PVAT(+)	18.0 ± 1.9 (9)	21.0 ± 2.3 (7)
Nebivolol E(+) /PVAT(−)	28.0 ± 2.4 (6)	27.9 ± 3.3 (6)
Nebivolol E(−) /PVAT(+)	22.4 ± 1.9 (6)	21.0 ± 2.5 (6)
Nebivolol E(−) /PVAT(−)	41.0 ± 4.3 (9)	39.6 ± 6.5 (6)

Abdominal aorta		
Groups	Control	Obese
Control E(+) /PVAT(+)	17.9 ± 2.4 (9)	30.2 ± 5.4 (6)^a^
Control E(+) /PVAT(−)	20.3 ± 2.8 (9)	21.8 ± 2.8 (7)
Control E(−) /PVAT(+)	22.6 ± 2.1 (7)	20.4 ± 2.5 (9)
Control E(−) /PVAT(−)	26.4 ± 3.8 (6)	27.8 ± 5.6 (9)
Nebivolol E(+) /PVAT(+)	20.0 ± 2.4 (7)	18.4 ± 2.0 (6)
Nebivolol E(+) /PVAT(−)	24.1 ± 1.4 (9)	21.3 ± 2.0 (8)
Nebivolol E(−) /PVAT(+)	19.3 ± 2.1 (9)	18.9 ± 2.8 (7)
Nebivolol E(−) /PVAT(−)	28.4 ± 3.3 (9)	28.8 ± 3.8 (7)

*Note:* The number (n) of animals used in each set of experiments is indicated after each set of measurements. Values are expressed as (mN) × log [mol/L] and given as means ± SEM.

^a^ Compared to the respective control, nebivolol and obese‐nebivolol group (*p* < 0.05; two‐way ANOVA followed by Tukey's post hoc test).

**FIGURE 6:**
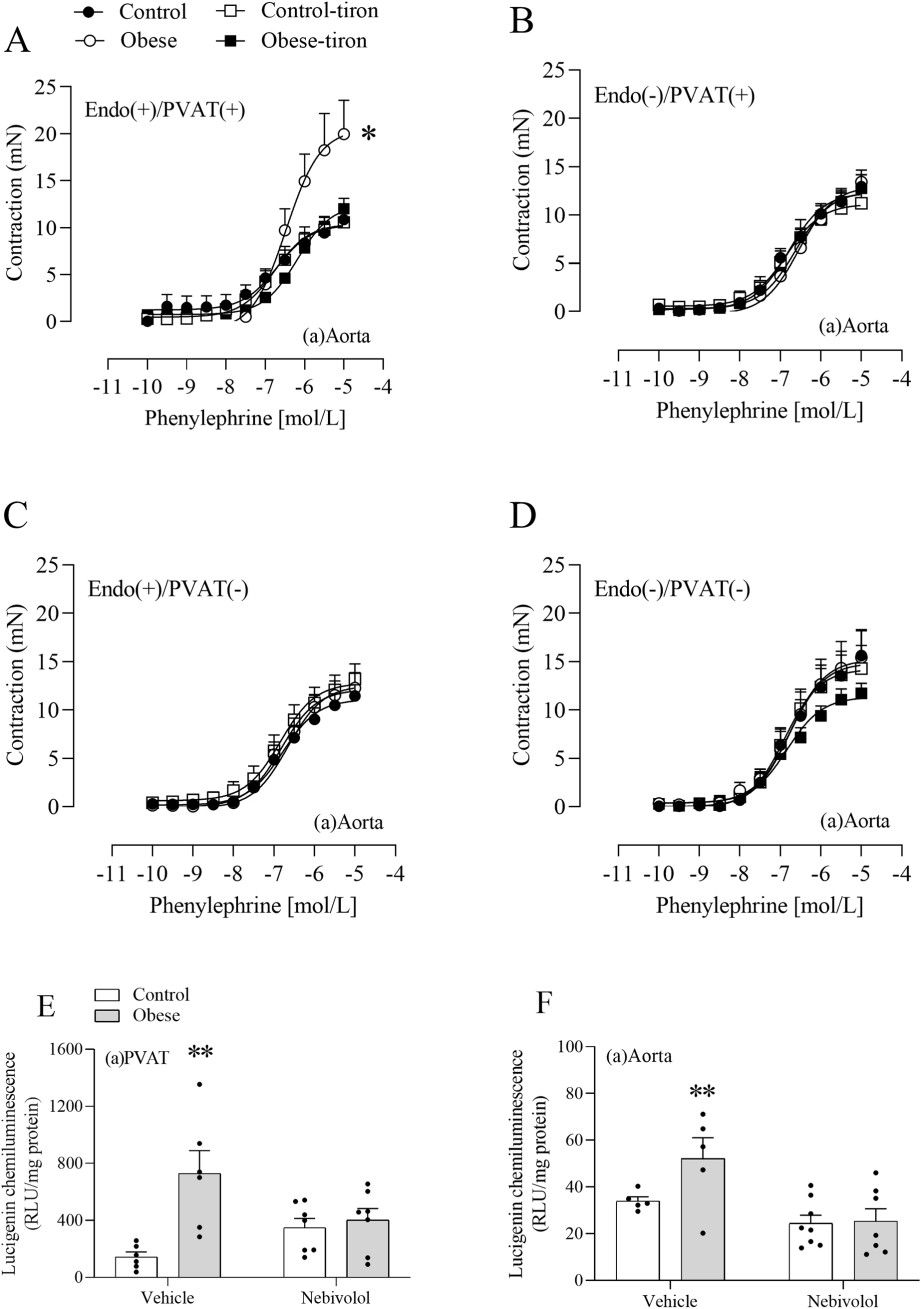
Tiron reverses obesity‐induced hypercontractility in the abdominal aorta. Concentration‐response curves to phenylephrine were obtained in the abdominal aorta [(a) Aorta] with intact [Endo(+)] or denuded endothelium [Endo(−)] in the presence [PVAT(+)] or absence of PVAT [PVAT(−)] after incubation (30 min) with tiron (1 mmol/L) (A–D). NADPH oxidase‐derived O_2_•^−^ was determined in the (a) Aorta and its PVAT [(a) PVAT] using the lucigenin chemiluminescence assay (E, F). Values are given as means ± SEM of *n* = 6–9 rats. *Compared to the control, control‐tiron, and obese‐tiron groups;**Compared to the control, nebivolol and obese‐nebivolol groups (*p* < 0.05; two‐way ANOVA followed by Tukey's post hoc test). The asterisk (*) denotes significant differences in either the E_max_ or pD_2_ values (provided in Table 2).

Tiron reversed obesity‐induced hypercontractility in endothelium‐intact arteries with PVAT (Figure 6A; Tables 2 and 4). However, it did not alter phenylephrine‐induced contraction in endothelium‐denuded arteries with PVAT (Figure 6B; Tables 2 and 4). In arteries without PVAT, whether endothelium‐intact or denuded, tiron incubation caused no changes in contraction (Figure 6C,D; Table 2). Obesity increased O_2_•^−^ generation in the abdominal aorta and PVAT, a response reversed by nebivolol (Figure 6E,F).

**TABLE 4:** Area under the curve (AUC) of the concentration response curves for phenylephrine in the absence or presence of tiron or L‐NAME.

Thoracic aorta		
Groups	Control	Obese
Control E(+) /PVAT(+)	15.5 ± 1.6 (9)	30.2 ± 4.2 (8)^a^
Control E(+) /PVAT(−)	25.3 ± 2.0 (9)	47.2 ± 3.4 (9)^a^
Control E(−) /PVAT(+)	20.4 ± 2.1 (9)	25.3 ± 5.2 (8)
Control E(−) /PVAT(−)	33.9 ± 3.0 (9)	32.8 ± 3.8 (9)
Tiron E(+) /PVAT(+)	14.8 ± 2.4 (9)	15.7 ± 1.3 (7)^b^
Tiron E(+) /PVAT(−)	28.8 ± 4.3 (8)	29.3 ± 3.7 (6)^b^
Tiron E(−) /PVAT(+)	25.6 ± 3.8 (9)	19.3 ± 3.1 (6)
Tiron E(−) /PVAT(−)	39.1 ± 5.5 (8)	28.7 ± 5.0 (9)
L‐NAME E(+) /PVAT(+)	31.2 ± 3.1 (5)^a^	32.6 ± 1.8 (6)
L‐NAME E(+) /PVAT(−)	41.8 ± 2.6 (7)^a^	41.0 ± 3.0 (5)

Abdominal aorta		
Groups	Control	Obese
Control E(+) /PVAT(+)	17.9 ± 2.4 (9)	30.2 ± 5.4 (6)^a^
Control E(+) /PVAT(−)	20.3 ± 2.8 (9)	21.8 ± 2.8 (7)
Control E(−) /PVAT(+)	22.6 ± 2.1 (7)	20.4 ± 2.5 (9)
Control E(−) /PVAT(−)	26.4 ± 3.8 (6)	27.8 ± 5.6 (9)
Tiron E(+) /PVAT(+)	19.8 ± 3.1 (8)	17.8 ± 1.5 (6)^b^
Tiron E(+) /PVAT(−)	25.5 ± 3.6 (7)	20.1 ± 2.2 (6)
Tiron E(−) /PVAT(+)	22.3 ± 3.1 (6)	21.1 ± 2.8 (7)
Tiron E(−) /PVAT(−)	26.8 ± 4.4 (7)	22.1 ± 2.2 (6)

*Note:* The number (n) of animals used in each set of experiments is indicated after each set of measurements. Values are expressed as (mN) × log [mol/L] and given as means ± SEM.

^a^ Compared to the control group.

^b^ Compared to the obese group in the absence of the inhibitor (*p* < 0.05; two‐way ANOVA followed by Tukey's post hoc test).

**FIGURE 7:**
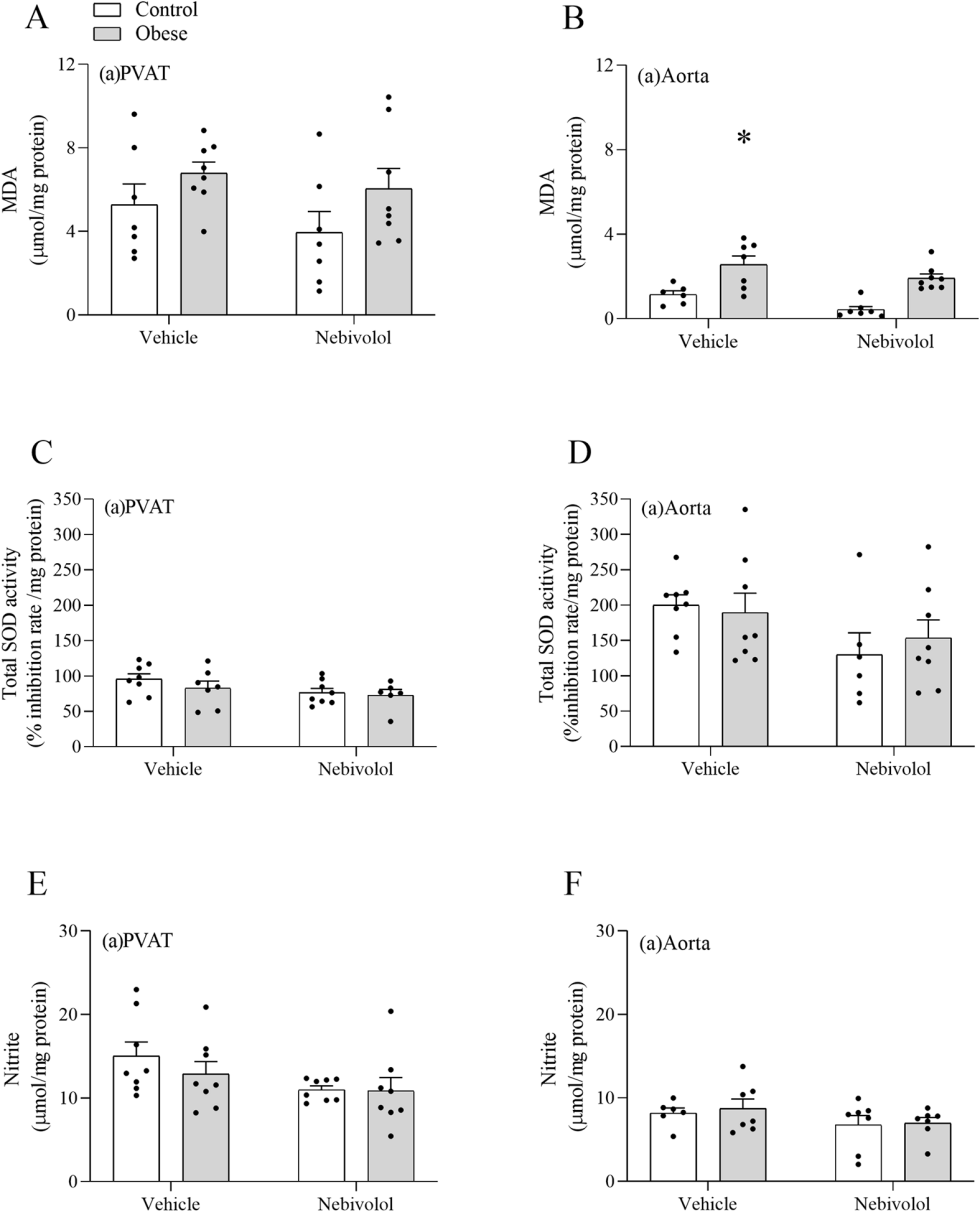
Effects of obesity on total SOD activity, lipoperoxidation and nitrite concentration in the abdominal aorta and abdominal PVAT. The concentration of MDA (A, B), the activity of SOD (C, D), and the concentration of nitrite (E, F) were determined colorimetrically. Values are given as means ± SEM of *n* = 6–8 rats. *Compared to the control, nebivolol, and obese‐nebivolol groups (*p* < 0.05; two‐way ANOVA followed by Tukey's post hoc test).

Obesity elevated the concentration of MDA in the abdominal aorta, but not in the PVAT. Nebivolol reversed obesity‐induced lipoperoxidation in aortas from obese rats (Figure 7A,B). However, obesity did not alter SOD activity (Figure 7C,D) or nitrite concentration (Figure 7E,F) in either the abdominal aorta or PVAT.

## 4 Discussion

Our findings confirm earlier studies showing that obesity is associated with vascular hypercontractility [8, 13]. Corroborating previous reports, we found that in thoracic aortas from control rats, PVAT attenuated the contractile response induced by phenylephrine in both endothelium‐intact and endothelium‐denuded preparations [17, 18]. An increased phenylephrine‐induced contraction was observed in endothelium‐intact aortas from obese rats, regardless of the presence of PVAT, indicating that obesity did not impair the anticontractile effect of (t)PVAT. Possible explanations for the unaffected PVAT function in obesity include diet composition and the duration of treatment. For example, a comparison of different obesogenic diets in rats revealed distinctive patterns of effects on oxidative stress, inflammatory markers, blood pressure, and lipid profile [28, 29]. Regarding the duration of treatment, it has been shown that long‐term feeding with obesogenic diets promotes cardiovascular changes that are not seen after shorter treatment periods [30].

Our functional studies in the thoracic aorta revealed that obesity‐induced hypercontractility occurred exclusively in arteries with an intact endothelium (regardless of PVAT presence). This finding suggests a potential link between obesity and endothelial dysfunction. However, while suggestive, our data are not conclusive, as they rely solely on functional responses to phenylephrine. The use of vasoactive agents that elicit endothelium‐dependent responses, such as acetylcholine, would help clarify whether the observed hypercontractility to phenylephrine is indeed attributable to endothelial dysfunction. This approach has been successfully employed in previous studies demonstrating that obesity impairs endothelium‐dependent relaxation induced by acetylcholine [7]. Therefore, the absence of functional assessments of endothelium‐dependent responses represents a limitation of the present study.

The overproduction of O_2_•^−^ is a critical mechanism underlying vascular hypercontractility in obesity. In this scenario, NADPH oxidase is a major source of O_2_•^−^ [31, 32]. Functionally, we found that tiron (a scavenger of O_2_•^−^) abrogated the hypercontractility to phenylephrine in the thoracic aorta from obese rats, implicating O_2_•^−^ in this response. Correspondingly, we observed that obesity elevated the levels of NADPH‐oxidase‐derived O_2_•^−^ in the thoracic aorta, suggesting an increased activity of this enzyme. This increase in O_2_•^−^ levels occurred alongside unchanged SOD activity. The primary function of the three oxidoreductases that compose the SOD family (SOD1–3) is to control O_2_•^−^ levels by promoting its conversion into H_2_O_2_ and O_2_ [33]. Consequently, alterations in SOD activity are directly linked to changes in O_2_•^−^ levels [34]. The fact that obesity did not alter SOD activity in the thoracic aorta reinforces the conclusion that the increase in O_2_•^−^ levels is due to its overproduction by the NADPH oxidase, rather than a decrease in its elimination by SOD.

In the rat thoracic aorta, NO is a crucial relaxing factor produced by endothelial cells [35, 36]. Consequently, a reduction in NO within this artery is typically associated with vascular hypercontractility [37, 38]. The bioavailability of NO can be diminished under conditions characterized by elevated levels of O_2_•^−^. This occurs because O_2_•^−^ reacts with NO to form peroxynitrite (ONOO^−^), a reactive secondary oxidizing species. The generation of ONOO^−^ has two major consequences: impaired vascular relaxation and increased lipoperoxidation [15, 39, 40]. In our study, obesity increased lipoperoxidation in the thoracic aorta, a response potentially mediated by ONOO^−^. However, our findings do not rule out the possibility that lipoperoxidation is directly mediated by O_2_•^−^

Given that O_2_•^−^ reduces NO bioavailability, we next assessed its impact on vascular contraction. Functionally, L‐NAME (an inhibitor of NO synthase) increased phenylephrine‐induced contraction in thoracic arteries with intact endothelium from control rats, but not from obese rats. Correspondingly, decreased nitrite concentrations (a stable metabolite of NO) were detected in the thoracic aorta of obese rats, suggesting reduced NO levels. Thus, our findings indicate that O_2_•^−^ diminishes NO bioavailability in the thoracic aorta, leading to increased vascular contraction.

Changes in the (t)PVAT of obese rats were identified, including increased production of NADPH‐oxidase‐derived O_2_•^−^, lipid peroxidation, elevated SOD activity, and reduced nitrite levels. However, despite these obesity‐induced alterations, (t)PVAT was not found to be dysfunctional. These changes did not affect vascular contractility, as demonstrated by our functional findings in the presence of (t)PVAT.

Nebivolol reversed obesity‐induced vascular hypercontractility in the thoracic aorta. The drug also reversed lipoperoxidation and the increase in lucigenin‐derived luminescence in arteries from obese rats, suggesting an inhibitory effect on NADPH oxidase activity. Moreover, nebivolol restored nitrite levels. Inhibition of NADPH oxidase activity is a central mechanism whereby nebivolol exerts vasculoprotective effects [24, 41, 42]. Thus, we suggest that nebivolol reversed the overproduction of O_2_•^−^ in obesity by inhibiting NADPH oxidase activity. By reducing O_2_•^−^ generation, nebivolol prevents NO inactivation by O_2_•^−^, thereby preserving NO levels and diminishing the generation of ONOO−. Consequently, nebivolol induces normalization of vascular contractility and a reduction in lipoperoxidation. Lastly, beneficial effects of nebivolol in the (t)PVAT were identified. As seen in the thoracic aorta, nebivolol reduced O_2_•^−^ generation, and lipoperoxidation and restored nitrite levels. The protective effects of nebivolol in the (t)PVAT are likely associated with its inhibition of NADPH oxidase activity, as the drug reduced lucigenin‐derived luminescence in the (t)PVAT. However, the effect of nebivolol in restoring NO levels must be interpreted with caution, as it was based on the concentration of nitrite. Nitrite measurement provides only an indirect and static snapshot of NO metabolism; it does not reflect real‐time NO production, eNOS activity, or NO consumption, particularly under conditions of elevated oxidative stress. Functional assays in aortas from obese rats treated with nebivolol, in the presence of L‐NAME, would be valuable for assessing the contribution of the nitrergic pathway in this study. Further experiments are needed to deepen the understanding of obesity pathophysiology and the mechanism of action of nebivolol.

Regarding the abdominal aorta, our functional findings showed that PVAT does not modulate phenylephrine‐induced contraction under physiological conditions, which is in agreement with previous observations [17]. Obesity‐induced dysfunction of the (a)PVAT was characterized by increased vascular contraction. Interestingly, (a)PVAT dysfunction occurred in an endothelium‐dependent manner. In arteries without PVAT, obesity did not alter phenylephrine‐induced contraction, which strengthens the proposal that the hypercontractility detected in arteries with PVAT from obese rats occurs due to a dysfunctional PVAT. Tiron reversed the vascular hypercontractility in arteries with (a)PVAT, suggesting the participation of O_2_•^−^ in obesity‐induced (a)PVAT dysfunction. Corroborating this finding, an increased generation of NADPH‐derived O_2_•^−^ was detected in the (a)PVAT from obese rats. This increased O_2_•^−^ concentration was not the result of reduced elimination by SOD, as no change in the activity of this enzyme was detected in the (a)PVAT of obese rats. Unlike what was observed in the (t)PVAT, obesity did not induce changes in nitrite concentration in the (a)PVAT. Of note, despite the augmented levels of O_2_•^−^ and lipoperoxidation in the abdominal aorta of obese rats, no changes in vascular contractility were observed in arteries without PVAT.

The exact mechanism by which obesity favors a pro‐contractile phenotype of (a)PVAT is uncertain. Physiologically, (a)PVAT does not exert anticontractile effects [17]. Therefore, the endothelium‐dependent dysfunction of (a)PVAT in obesity described here is not the result of a decrease in PVAT‐derived vasorelaxant factors. In fact, we demonstrated that this PVAT dysfunction is mediated by O_2_•^−^. Consequently, we propose that endothelium‐derived O_2_•^−^ triggers the release or production of PVAT‐derived vasocontractile factors, which ultimately mediate hypercontractility to phenylephrine. This idea is consistent with previous observations showing that O_2_•^−^ stimulates the production of the vasocontractile prostanoids thromboxane A_2_ and prostaglandin F_2_α within PVAT, resulting in a shift to a pro‐contractile profile and vascular hypercontractility [43, 44]. Nebivolol reversed the pro‐contractile phenotype of (a)PVAT induced by obesity, possibly by reducing the overproduction of O_2_•^−^ by NADPH oxidase. Additionally, the reduction in O_2_•^−^ levels in the abdominal aorta may account for its protective effect, since (a)PVAT dysfunction is endothelium‐dependent.

## 5 Conclusion

**FIGURE 8:**
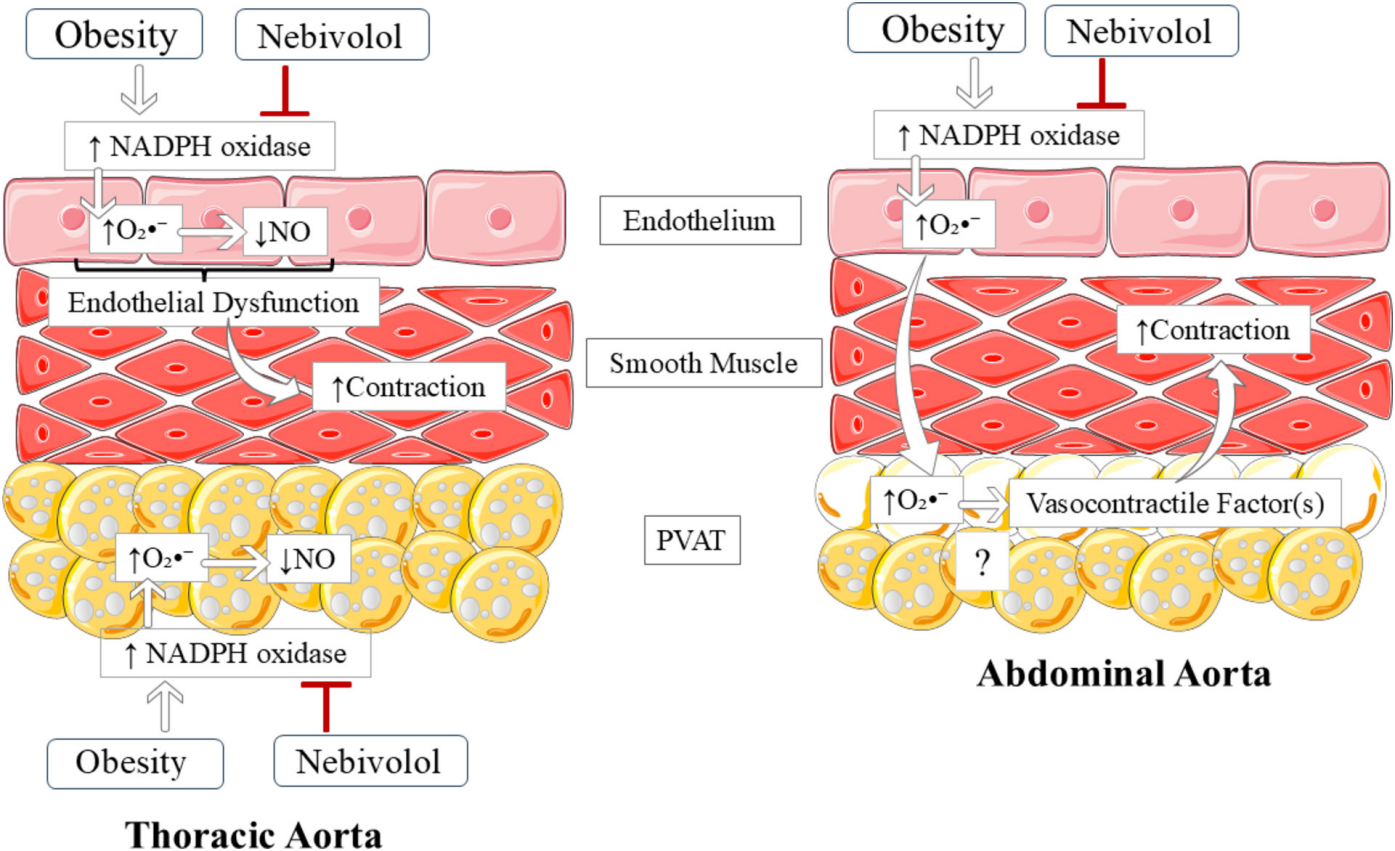
Proposed vasculoprotective mechanism of nebivolol on obesity‐induced dysfunction of the thoracic and abdominal aorta. Thoracic aorta: obesity induces endothelial dysfunction associated with the overproduction of O_2_•^−^ and reduced NO bioavailability, resulting in hypercontractility. No change in the anticontractile effect of thoracic PVAT was detected. Nebivolol reversed the endothelial dysfunction by inhibiting NADPH oxidase activity and O_2_•^−^ generation. Abdominal aorta: obesity induces a pro‐contractile phenotype of the abdominal PVAT via an endothelium‐dependent mechanism mediated by O_2_•^−^. Nebivolol restored PVAT function by inhibiting NADPH oxidase activity and O_2_•^−^ generation. NO: nitric oxide; O_2_•^−^: superoxide; PVAT: perivascular adipose tissue; (?): uncertain mechanism.

The novelty of this study lies in demonstrating that obesity differentially affects the thoracic and abdominal aorta along with their associated PVATs. Furthermore, we provide the first evidence that nebivolol exerts vasculoprotective effects via antioxidant mechanisms within the PVAT of conduit arteries, ultimately reversing obesity‐induced vascular hypercontractility in both aortic segments (Figure 8). The (a)PVAT assumes a pro‐contractile phenotype that is endothelium‐dependent and involves the overproduction of O_2_•^−^. In contrast, in the (t)PVAT, obesity promotes pro‐oxidative effects that do not result in a loss of its inherent anticontractile function. The hypercontractility of the thoracic aorta in obesity is PVAT‐independent, whereas changes in the abdominal aorta are observed solely in the presence of its PVAT. Nebivolol displays vasculoprotective effects through antioxidant mechanisms, leading to the reversal of vascular hypercontractility in both aortic segments. Drugs that promote such beneficial vascular effects could help mitigate cardiovascular complications associated with obesity.

## Author Contributions

T.M.H.D.: conceptualization, methodology, editing, data analyses, writing; G.F.P., L.P.S., V.O.A., and A.O.S.: data curation, data analyses. C.R.T.: conceptualization, supervision, writing. All authors read and approved the manuscript. The authors declare that all data were generated in‐house and that no paper mill was used.

## Funding

This work was supported by the Fundação de Amparo à Pesquisa do Estado de São Paulo (FAPESP, 2022/11304‐0) and the Coordenação de Aperfeiçoamento de Pessoal de Nível Superior (CAPES, Finance Code 001).

## Ethics Statement

Housing conditions and experimental protocols followed the guidelines of the National Research Council's Guide for the Care and Use of Laboratory Animals (8th edition) and the National Committee for Animal Experimentation Control (CONCEA, Brazil) and were approved by the local Ethics Committee on Animal Use (protocol No. 21.1.304.22.3). All animal studies comply with the ARRIVE guidelines.

## Consent

The authors have nothing to report.

## Conflicts of Interest

The authors declare no conflicts of interest.

## Data Availability

Data are provided within the manuscript.
